# High Frequency of Intravenous Injection of Human Adipose Stem Cell Conditioned Medium Improved Embryo Development of Mice in Advanced Maternal Age through Antioxidant Effects

**DOI:** 10.3390/ani10060978

**Published:** 2020-06-04

**Authors:** Kihae Ra, Hyun Ju Oh, Geon A Kim, Sung Keun Kang, Jeong Chan Ra, Byeong Chun Lee

**Affiliations:** 1 Department of Theriogenology and Biotechnology, College of Veterinary Medicine, Seoul National University, Seoul 08826, Korea; ragh1102@naver.com (K.R.); jooya5@snu.ac.kr (H.J.O.); 2 Department of Biomedical Laboratory Science, School of Medicine, Eulji University, Daejeon 34824, Korea; pshsje03@snu.ac.kr; 3 Biostar Stem Cell Research Institute, R Bio Co., Ltd., Seoul 08506, Korea; kangsk@stemcellbio.com (S.K.K.); jcra@stemcellbio.com (J.C.R.)

**Keywords:** adipose stem cell, advanced maternal age, conditioned medium, embryo development, intravenous injection

## Abstract

**Simple Summary:**

In this study, we examined the anti-oxidative effect of human adipose stem cell conditioned medium (ASC-CM) on the ovary and uterus of mice in advanced maternal age (AMA) and the optimal conditions of intravenous injection for ASC-CM administration. Human ASC-CM upregulated expression of antioxidant genes, restored the quality of oocytes derived from aged ovaries and resulted in improved in vitro and in vivo embryo development. The anti-oxidative effect human ASC-CM was optimized with high frequency of administration. Comprehensively, our study successfully introduced the potential of ASC-CM as an antioxidant intervention against age-related infertility in AMA.

**Abstract:**

Advanced maternal age (AMA) has become prevalent globally. With aging, weakened antioxidant defense causes loss of normal function in the ovary and uterus due to oxidative stress. Here, we aimed to improve embryo development in AMA mice by intravenous injection (IV) of human adipose stem cell conditioned medium (ASC-CM) at various frequencies and intervals as an antioxidant intervention. Four- and six-month-old female ICR (Institute of Cancer Research) mice were randomly divided into groups IV treated with human ASC-CM under different conditions, and in vitro and in vivo embryo development were evaluated. Consequently, compared to the control group, blastocyst formation rate of parthenotes was significantly promoted in 4-month-old mice and the mean number of implanted fetuses after natural mating was significantly increased by approximately two-fold in 6-month-old mice. Through gene analysis, the anti-apoptotic and anti-oxidative effects of human ASC-CMs were confirmed in the ovaries and uterus of pregnant mice at both ages. In particular, ovarian expression of *gpx1* and catalase drastically increased in 6-month-old mice. Furthermore, the levels of *gpx1* and catalase were further increased, with a high frequency of injection regardless of age. Thus, we demonstrated for the first time the anti-oxidative effect of human ASC-CM administration against ovarian aging and the optimal injection condition.

## 1. Introduction

During the past decades, the average child-bearing age of women has increased to over 30 and fertility rates in those aged over 35 years, regarded as advanced maternal age (AMA), have risen in most member countries of the organization for economic co-operation and development countries [1]. Fertility normally declines with increasing age and there is a growing risk of pregnancy failure in AMA; this has generated the need to develop and employ assisted reproductive technology (ART) [2]. Despite the recent developments of ART, there still exist limitations in pregnancy and live birth rate in AMA compared to younger ages [3]. The quality of oocytes and uterus receptivity are critical factors that affect successful embryo development and maintenance of pregnancy in ART; however, these factors degenerate as maternal age increases [4]. Not only the quantity, but also the quality of oocytes decreases with aging of the ovary [5], which commonly leads to the prevalence of chromosomal abnormalities in oocytes. Deterioration of oocyte quality may result in adverse outcomes including impaired embryo development, implantation failure, and miscarriage [6]. Reduction of endometrial receptivity in AMA, which is associated with a decrease in uterine blood flow and progesterone sensitivity [7], increases the chance of implantation failure [8]. Even after implantation, fetuses in AMA are exposed to a higher risk of premature growth and intrauterine death [9].

The reproductive disorders in the ovary and uterus described above may be due to oxidative stress, as reactive oxygen species (ROS) levels increase and anti-oxidative balance fails with aging [10]. Diverse antioxidant agents have been investigated to alleviate oxidative stress including adipose stem cells (ASCs). ASCs are mesenchymal stem cells derived from adipose tissue, which are used in regenerative medicine because they display stable differentiation ability and can be easily obtained in adequate amounts [11]. The secretory factors of ASCs affect various physiological processes including apoptosis, immunoregulation, and angiogenesis [12]. Furthermore, it has been shown that ASCs secrete antioxidant factors [13]. In practice, ASCs treatment has been used in cases of female infertility due to ovarian dysfunction, premature ovarian insufficiency, and Asherman syndrome [14]. Conditioned medium (CM), defined as medium obtained from the source cell culture under certain conditions, has been recently confirmed to contain the secretory factors of ASC and their anti-oxidative effects [13]. The therapeutic ability of CM has been assessed to be comparable with that of conventional cell transplantation and as a derivative not containing the original living cells, the reliability and reproducibility of CM treatment can be assured [15]. Furthermore, CM can be more applicable compared to cells because it can be produced in high amounts, transported, and stored as a liquid, and most importantly, it does not trigger cell donor–recipient immune rejection [16].

Stem cells can be administered systemically or locally through different delivery routes. Local administration enables the injected stem cells to act immediately on the targeted site, but surgical intervention is required, which may lead to traumatic damage [17]. Among the systemic administration methods, intravenous injection (IV) is most commonly applied for the delivery of stem cells and its secretory factor with minimal invasion [17]. Notably, clinical improvements were observed after IV of ASCs [18], and its safety regarding toxicity and tumorigenicity has been verified [11]; however, the optimal dosing intensity and frequency should be determined to ensure its therapeutic efficacy, which varies depending on each case [19]. To the best of our knowledge, no study has reported on the antioxidant effects of ASC-CM IV administration frequency on the reproductive competence in AMA. In the present study, we hypothesized that intravenously administered human ASC-CMs could attenuate oxidative stress in the ovary and uterus and consequently, enhance embryo development in AMA mice, depending on the injection conditions. The aim of this study was (1) to determine the optimal IV conditions of human ASC-CMs for improving in vitro and in vivo embryo development in AMA mice, and (2) to investigate the expression of genes on oxidative stress (*sod2* [20], *gpx1* [21], catalase [22]) and apoptosis (*bax* [23], *bcl2* [24], *caspase3* [25]) to evaluate the anti-oxidative effect of human ASC-CM IV.

## 2. Materials and Methods

### 2.1. Ethics Approval

Human ASC-CMs were provided by the R Bio Stem Cell Research Center under good manufacturing practice conditions. All cell donors provided informed consent to participate in the study. The research was approved by the Life Ethics Committee of Biostar Stem Cell Technology (RBIO 2015-12-001). The details of specific standards are found in the Code of Federal Regulations, Title 21 (21CFR), and Section 610.

### 2.2. Chemicals

All chemicals and reagents were purchased from Sigma-Aldrich (St. Louis, MO, USA) unless otherwise stated.

### 2.3. Isolation of Human Adipose Stem Cell and Preparation of Conditioned Medium

Adipose tissue was collected from a 39-year-old woman by the subcutaneous liposuction method after eligibility determination for donors of human cells, tissues, and cellular and tissue-based products. Adipose tissue-derived stem cells were isolated using a previously described process [11] and stored in a liquid nitrogen tank. For immunophenotypic characterization, ASCs suspended in phosphate buffered saline (PBS) were labeled and incubated with antibodies against positive and negative markers of MSC for 30–60 min. The expression of CD31-FITC, CD34-FITC, CD45-FITC, CD73-PE, and CD90-PE, the surface markers for the identification of MSC [26] and in specific ASC [27], was analyzed by flow cytometry using a BD FACSCalibur™ flow cytometer and CellQuest Pro software (BD Biosciences, San Jose, CA, USA).

To collect conditioned medium, ASCs were thawed in a T-175 flask (175cm^2^) with AMSC medium for adipose tissue-derived stem cell culture (R BIO, Seoul, Korea) at 37 °C and 5% CO_2_. A total of 3 × 10^7^ of ASCs were sub-cultured into the hyper flask and cultured with AMSC medium for 48 h, then replaced with serum-free Dulbecco’s Modified Eagle’s Medium (DMEM; Invitrogen, Grand Island, NY, USA). The culture medium was collected after 24 h and replaced with fresh medium; this was repeated five times. The total CM collected over five days were centrifuged at 2500 rpm for 5 min, mixed, and processed for sterilization and filtration using a 0.22 μm filter.

### 2.4. Animals and Treatments

All procedures with experimental animals were approved by the Institutional Animal Care and Use Committee of Seoul National University (SNU-170511-2-4) and designed to minimize the number of animals used and any suffering caused by the study. Briefly, 4- and 6-month-old AMA female and 8- to 12-week-old male ICR (Institute of Cancer Research) mice were purchased from Japan SLC, Inc. (Shizuoka, Japan). They were housed under controlled temperature and humidity (23 °C, 60%) with a 12 h light/dark cycle in a specific pathogen-free animal facility. Female mice at a certain age were randomly divided into control and treatment groups; the treatment group was administered IV of human ASCs via the tail vein three times with eight day intervals (3T-8D) and six times with four day intervals (6T-4D). Phosphate buffered saline was intravenously injected into the age-matched control group and the single dose amount was determined based on the weight of each mouse (1 μL/g) in all groups. The female mice in each group were used for parthenogenetic activation of oocytes and natural mating and were consequently evaluated for in vitro and in vivo embryo development (Supplementary Tables S1 and S2).

### 2.5. Oocyte Collection

On the day of the last IV, superovulation of female mice in each group was induced by intra-peritoneal injection of hormones with 7.5 IU pregnant mare serum gonadotropin (Calbiochem, La Jolla, CA, USA), followed by 7.5 IU human chorionic gonadotropin (hCG) 46–48 h later. Mice were humanely sacrificed by cervical dislocation and the ovaries were collected approximately 14 h post-hCG injection.

Cumulus-oocyte complexes were collected from the oviduct ampullae and the cumulus cells were denuded by brief incubation in M2 medium containing 0.1% hyaluronidase. Oocytes were gently washed in M2 medium and then the total number of retrieved oocytes and viable oocytes for parthenogenetic activation were counted by morphology assessment using a stereomicroscope.

### 2.6. Parthenogenetic Activation and Evaluation of Parthenote Development

Oocytes were washed and stabilized in potassium simplex optimized medium (KSOM; MTI global stem, Rockville, MD, USA) for 30 min in a humidified incubator at 36 °C with 5% CO_2_. Then, oocytes were activated by incubation in KSOM supplemented with 10 mM SrCl2, 5 mM cytochalasin B, and 2 mM EGTA for 1 h. Finally, parthenogenetically activated embryos were washed at least three times and in vitro cultured (IVC) in fresh KSOM drop. The cleavage rate of parthenotes was evaluated two days post-IVC, and the rate of blastocyst formation was evaluated six days post-IVC using a stereomicroscope. The total cell number per blastocyst was counted under a fluorescence microscope (Nikon Corp., Tokyo, Japan) by staining with 5 µg/mL Hoechst 33342 for 10 min.

### 2.7. Assessment of Natural Mating and Implantation

Estrous cycles of female mice in each group was identified by visual observation of their vagina approximately two weeks before the day of the last IV. A female mouse or two mice with synchronized estrous cycles were mated with an intact male mouse on the nearest pro-estrous day after the last IV. Female mice were separated in a new cage upon observation of a vaginal plug on the following morning and considered to be pregnant. Six days after the detection of the vaginal plug, female mice were humanely sacrificed by cervical dislocation. From both ovaries to uterine cervix were excised for RNA isolation and the fetus-placenta complex was isolated from the uterine horns to evaluate the number of implanted fetuses.

### 2.8. Gene Analysis by Quantitative Real-Time Polymerase Chain Reaction

Total RNA was extracted from the ovaries and uterine tissues collected from pregnant mice of each group using the easy-spin Total RNA Extraction Kit (iNtRON, Gyeonggi, Korea), according to the manufacturer’s protocol. The concentration of total RNA was measured using a NanoDrop 2000 Spectrophotometer (Thermo Fisher Scientific, Wilmington, DE, USA, Supplementary Table S2). Complementary DNA (cDNA) was synthesized using a Maxime RT premix kit (iNtRON). The real-time PCR plate was reacted on a StepOnePlus Real-Time PCR System (Applied Biosystems, Waltham, MA, USA) with a final volume of 20 μL, containing 0.4 μL forward primer, 0.4 μL reverse primer, 10 μL SYBR Green PCR master mix (Applied Biosystems), 7.2 μL diethylpyrocarbonate treated water, and 1 μL cDNA. Real-time polymerase chain reaction (PCR) was carried out under the following conditions: 95 °C for 10 min, 95 °C for 10 s, 60 °C for 20 s, and 40 cycles of 72 °C for 40 s; at least three replications were performed. The expression of each target gene was quantified relative to the expression of the control house-keeping gene *18S rRNA* [28]. Relative quantification was based on the comparison of ΔCt values at constant fluorescence intensity. The relative formula (R) was calculated using the formula: R = 2^- [ΔCt sample-ΔCt control group]^; each obtained value was normalized to the value of *18S rRNA* and the average value for each gene expression of control group was set as one. The primer sequences used in this study are shown in Table 1.

### 2.9. Statistical Analysis

Statistical analyses were performed using one-way analysis of variance with Tukey’s post-test in GraphPad Prism software version 5 (GraphPad, San Diego, CA, USA). Experiments were repeated at least three times. Data are presented as means ± standard error of the mean. *p*-Values < 0.05 among the groups were considered statistically significant.

## 3. Results

### 3.1. Characterization of Isolated ASCs

**Figure 1:**
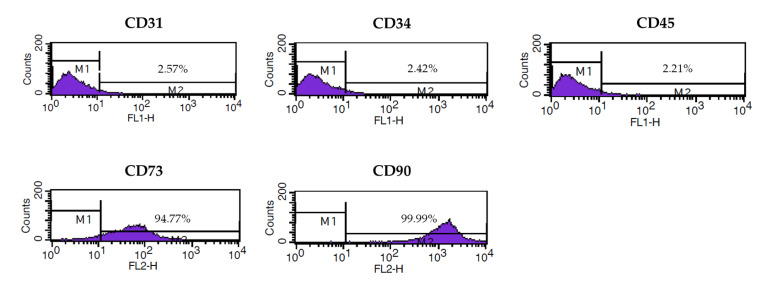
Characterization of human adipose-derived stem cells (ASCs) The surface markers were analyzed by flow cytometry in ASCs with positive expression of CD73 and CD90 but negative for CD31, CD34, and CD45.

The expression of cell surface markers in ASCs isolated from a healthy female donor was analyzed by flow cytometry. The mesenchymal stem cell-specific markers CD73 and CD90 were expressed in the ASCs. In contrast, the endothelial cells, hematopoietic stem cells, and hematopoietic lineage markers, CD31, CD34, and CD45, respectively, were not expressed in the ASCs (Figure 1).

### 3.2. Ovulation and in Vitro Development of Parthenotes from 4- and 6-Month-Old Mice

**Figure 2:**
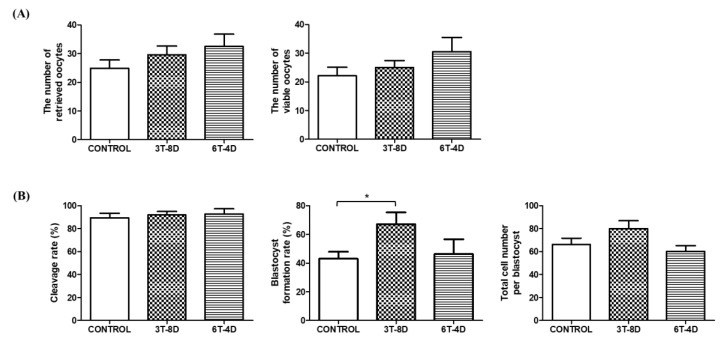
Evaluation of (**A**) ovulation status and (**B**) embryo development competence after parthenogenetic activation from the control and groups of human adipose-derived stem cell conditioned medium (ASC-CM) intravenous injection in 4-month-old mice. Data are expressed as the mean ± standard error of the mean (SEM). * *p* < 0.05. 4D-6T, six times injection in four day intervals; 8D-3T, three times injection in eight day intervals.

**Figure 3:**
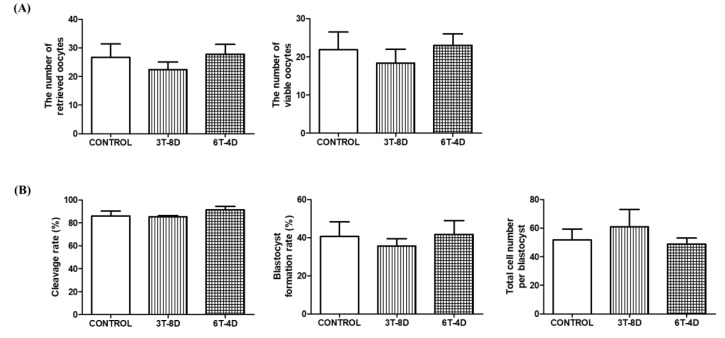
Evaluation of (**A**) ovulation status and (**B**) embryo development competence after parthenogenetic activation from the control and groups of human adipose-derived stem cell conditioned medium ASC-CM intravenous injection in 6-month-old mice. Data are expressed as the mean ± standard error of the mean (SEM). 4D-6T, six times injection in four day intervals; 8D-3T, three times injection in eight day intervals.

To identify the effect of human ASC-CMs on the quantity and quality of ovulated oocytes, the total number of oocytes after superovulation induction was counted. The number of oocytes available for parthenogenetic activation excluding fragmented or morphologically abnormal oocytes was assessed. As a result, the number of retrieved oocytes and viable oocytes were not significantly different among the control and treated groups in both 4- and 6-month-old mice (Figure 2A and Figure 3A). Parthenogenetic activation was conducted using viable oocytes and the cleavage rate and blastocyst formation rate of parthenotes were evaluated. In 4-month-old mice, the rate of cleaved embryos was similar among groups, but blastocyst formation rate in the 3T-8D group was significantly higher than the control group (67.01 ± 8.32 vs. 43.01 ± 4.91, *p* < 0.05, Figure 2B). Total cell number per blastocyst was not significantly different among the groups (Figure 2B). In the 6-month-old mice, there was no significant difference in cleavage rate, blastocyst formation rate, and total cell number per blastocyst among groups (Figure 3B).

### 3.3. Implanted Fetuses of 4- and 6-Month-Old Mice

**Figure 4:**
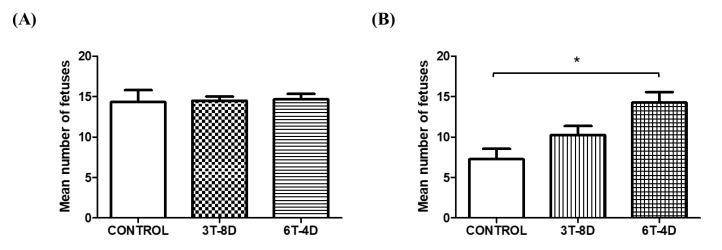
Assessment of fetus implantation after natural mating from control and groups of human adipose-derived stem cell conditioned medium (ASC-CM) intravenous injection in (**A**) 4-month-old and (**B**) 6-month-old mice. Data are expressed as the mean ± standard error of the mean (SEM). * *p* < 0.05. 4D-6T, six times injection in four day intervals; 8D-3T, three times injection in eight day intervals.

After natural mating with intact males, the number of implanted fetuses was evaluated six days after the vaginal plug formation to evaluate the in vivo development of embryos. Although a significant difference was not observed in the number of fetuses among groups in 4-month-old mice (Figure 4A), the number of fetuses in the 6T-4D group was significantly greater than in the control group (14.25 ± 1.32 vs. 7.25 ± 1.32, *p* < 0.05) in 6-month-old mice, but that in the 3T-8D group was not significantly different with the other groups (Figure 4B).

### 3.4. Anti-Apoptotic and Anti-Oxidative Effects of Human ASC-CMs in the Ovaries of Pregnant Mice

**Figure 5:**
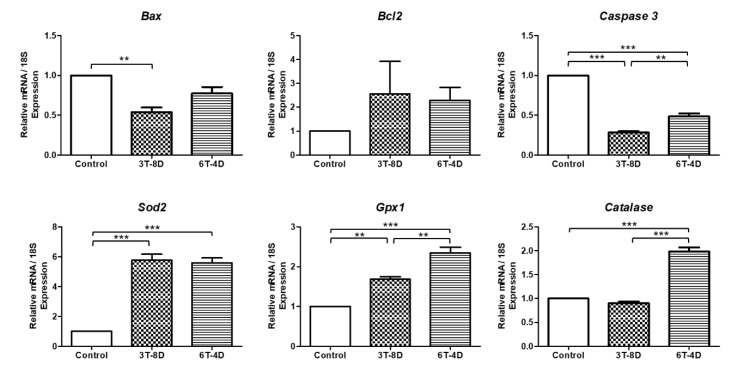
Relative expression to 18S of apoptosis and oxidative stress-related genes from the ovary of the control and groups of human adipose-derived stem cell conditioned medium (ASC-CM) intravenous injection to 4-month-old ICR mice. Data are expressed as the mean ± standard error of the mean (SEM). ** *p* < 0.01, *** *p* < 0.001. 4D-6T, six times injection in four day intervals; 8D-3T, three times injection in eight day intervals.

In 4-month-old mice, as shown in Figure 5, the relative expressions of the pro-apoptotic genes *Bax* and *Caspase3* were significantly decreased in the ovaries of the 3T-8D group than in the control group (*p* < 0.01, *p* < 0.001, respectively,) and *caspase* levels were significantly lower in the 6T-4D group than in the control group (*p* < 0.001), but higher than that in the 3T-8D group (*p* < 0.01). Moreover, the levels of *Bcl2* was higher in the 3T-8D and 6T-4D groups than in the control, but the difference among groups was not statistically significant. The expression of the antioxidant related gene *Sod2* was significantly higher in both the 3T-8D and 6T-4D groups than in the control group (*p* < 0.001). The level of *Gpx1* was higher in the 6T-4D group than in the control (*p* < 0.001) and 3T-8D groups (*p* < 0.01), and catalase was also expressed at higher levels in the 6T-4D group than in the control and 3T-8D groups (*p* < 0.001).

**Figure 6:**
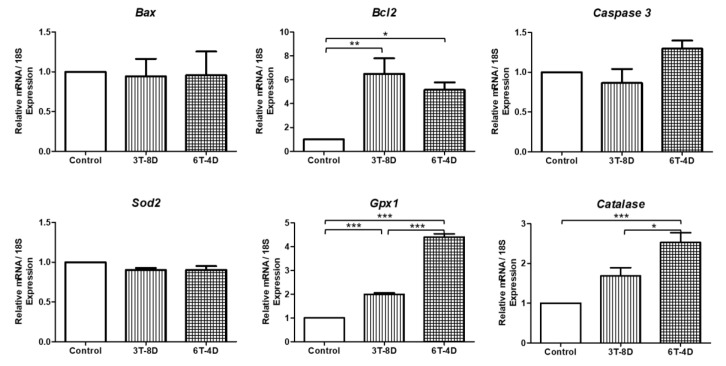
Relative expression to 18S of apoptosis and oxidative stress-related genes from the ovary of the control and groups of human adipose-derived stem cell conditioned medium (ASC-CM) intravenous injection to 6-month-old ICR mice. Data are expressed as the mean ± standard error of the mean (SEM). * *p* < 0.05, ** *p* < 0.01, *** *p* < 0.001. 4D-6T, six times injection in 4 four day intervals; 8D-3T, three times injection in eight day intervals.

Analysis of the relative gene expression in 6-month-old mice (Figure 6) revealed that the antiapoptotic gene *Bcl2* was significantly increased in both the 3T-8D and 6T-4D groups (*p* < 0.01, *p* < 0.05, respectively). *Bax* and *caspase 3* levels showed no difference among the groups. The level of *Sod2* was similar among the groups. *Gpx1* in 6-month-old mice demonstrated the same pattern of expression as 4-month-old mice in that both 3T-8D and 6T-4D groups showed significantly higher *Gpx1* levels than the control group (*p* < 0.001); in addition, the 6T-4D group exhibited significantly higher expression of *Gpx1* than the 3T-8D group (*p* < 0.001). The expression of catalase in the 6T-4D group was significantly higher than in the control and 3T-8D groups (*p* < 0.001 and *p* < 0.05, respectively).

### 3.5. Anti-Apoptotic and Anti-Oxidative Effects of Human ASC-CM in the Uterus of Pregnant Mice

**Figure 7:**
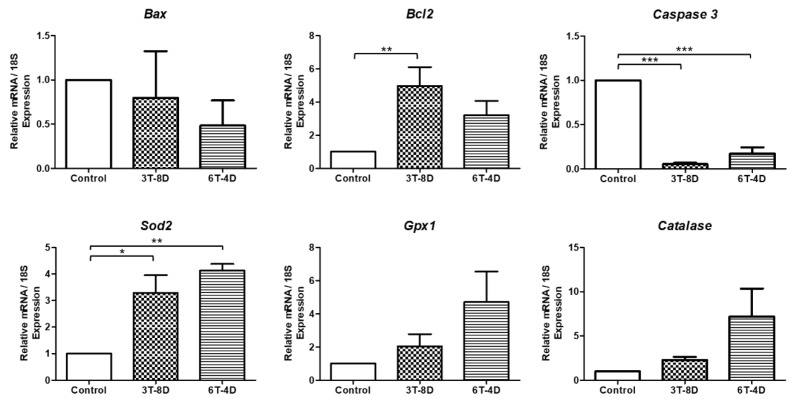
Relative expression to 18S of apoptosis and oxidative stress-related genes from the uterus of control and groups of human adipose-derived stem cell conditioned medium (ASC-CM) intravenous injection to 4-month-old ICR mice. Data are expressed as the mean ± standard error of the mean (SEM). * *p* < 0.05, ** *p* < 0.01, *** *p* < 0.001. 4D-6T, six times injection in four day intervals; 8D-3T, three times injection in eight day intervals.

In 4-month-old mice (Figure 7), *Bcl2* was significantly higher in the 3T-8D group than in the control group (*p* < 0.01). In contrast, a significant decrease in *Caspase 3* level was observed in both the 3T-8D and 6T-4D groups compared to the control (*p* < 0.001). There was no significant difference in *Bax* levels among the groups. With regard to anti-oxidative stress, *Sod2* expression was significantly higher in both the 3T-8D and 6T-4D groups compared to the control group (*p* < 0.05, *p* < 0.01, respectively,). *Gpx1* and catalase showed higher levels in the 3T-8D and 6T-4D groups than in the control group, but were not statistically significant among groups.

**Figure 8:**
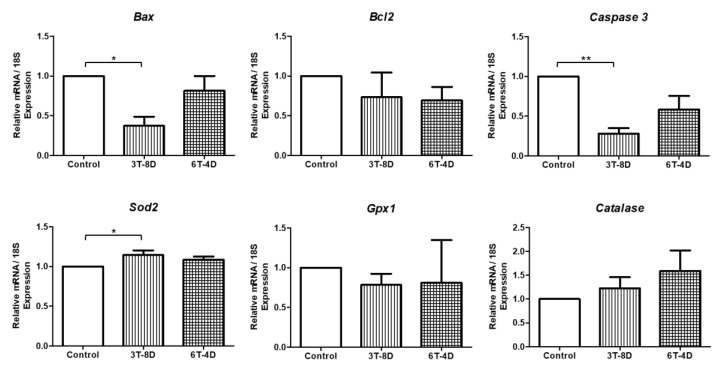
Relative expression to 18S of apoptosis and oxidative stress-related genes from the uterus of control and groups of human adipose-derived stem cell conditioned medium (ASC-CM) intravenous injection to 6-month-old ICR mice. Data are expressed as the mean ± standard error of the mean (SEM). * *p* < 0.05, ** *p* < 0.01. 4D-6T, six times injection in four day intervals; 8D-3T, three times injection in eight day intervals.

As shown in Figure 8, *Bax* and *Caspase 3* were significantly lower in the 3T-8D group than in the control group in 6-month old mice (*p* < 0.05, *p* < 0.01, respectively). *Bcl2* showed no significant difference in expression levels among the groups. There was a significant increase in *Sod2* level in the 3T-8D group compared to the control group (*p* < 0.05). *Gpx1* and catalase were expressed similarly among groups.

## 4. Discussion

To our knowledge, this is the first study to demonstrate the anti-oxidative effect of human ASC-CM, which enhances in vitro and in vivo embryo development with an increase in the anti-oxidative and anti-apoptotic gene expression, as confirmed in the ovary and uterus of AMA mice under optimal IV condition. For evaluation of in vitro embryo development, oocytes were collected from the control and treatment groups to perform parthenogenetic activation. Previous studies from our lab showed that the use of IV of human endothelial progenitor cells as an anti-aging agent could not alleviate age-related depletion of the oocyte pool [28]; no significant differences were identified in the numbers of retrieved oocytes and viable oocytes in either 4- or 6-month old mice. Despite preventive cell treatment for oocyte loss, a decrease in follicle pool with ovarian aging might not be rescued due to the influence of endocrine, paracrine, genetic, and metabolic factors [31].

Development of the blastocyst, which interacts with the uterus and determines the success of implantation [32], is an index for measuring the developmental potential of oocytes [33]. Considering that the developmental competence of oocytes is determined during ovary maturation [34], it is thought that the anti-oxidative effect of human ASC-CM improved the quality of oocytes in the aged ovaries and subsequently affected parthenote development during the blastocyst stage based on the results obtained, which indicated that the blastocyst formation rate of parthenotes was significantly increased in human ASC-CM-injected 4 month-old mice, but not at 6 months of age. Although both 4- and 6-month mice were considered as AMA in this study, it has been reported that fertility differences exist even within this AMA range as the reproductive efficiency of 6-month-old mice was significantly lower than that of the 4-month-old mice [35]. In addition to the relatively severe reproductive aging in 6-month-old mice, the external stress that in vitro manipulated embryos are exposed to should also be considered. Accordingly, it is presumed that although the anti-oxidative potential of human ASC-CM was able to overcome oxidative stress in in vitro developed embryos in younger AMA, its effect was not sufficient for preventing the formation of ROS due to intracellular and extracellular factors in older AMA.

Surprisingly, in contrast with the in vitro results, in vivo embryo development was enhanced in 6-month-old mice treated with high frequency of human ASC-CM IV. The mean numbers of implanted fetuses was increased two-fold compared to the control group, which is even higher than the average litter size of young commercial ICR mice [36]. The litter size is an intricate physiological element affected by sequential events including ovulation, fertilization, embryo development, and fetal survival [37]. Fan et al. reported that antioxidant administration decreased the oxidative stress on the implantation of fertilized embryos and fetal development in uterus during the early gestation period and subsequently improved the litter size [38], which is considered as an indicator of reproductive performance [39]. As a consequence, we analyzed variations in gene expression levels related to apoptosis and antioxidation in the ovaries and uterus of pregnant mice according to the group they were assigned to.

The anti-apoptotic and anti-oxidative effects of human ASC-CM were confirmed in the ovaries and uterus of mice of all ages with AMA. Age-related changes in both ovary and uterus reduced the probability of a successful pregnancy outcome [40]. Communication between the embryo and endometrium plays an important role in implantation, especially endometrial invasion into matured endometrium by the blastocyst [41]. Recent studies have demonstrated that aged patients using oocytes from younger donors achieved stable outcomes of embryo implantation and live birth regardless of their age, which suggested that pregnancy failures during AMA are closely associated with oocytes rather than uterine factors [42]. The present study demonstrated particularly remarkable results in ovaries where human ASC-CM IV increased the expression levels of *bcl2, gpx1,* and catalase by a maximum of approximately 6-, 4.5-, and 2.5-fold, respectively, in 6-month-old treated groups, whereas in 4-month-old mice, only 2-fold increases in *gpx1* and catalase levels were observed while no difference was observed in *bcl2* levels compared to the control group.

*Gpx1*, the most abundant form of *gpx*, is associated with female reproductive functions as it determines the growth and maturation of the follicles and follicular microenvironment [43]. It was reported that the activity of *gpx* was lower in postmenopausal ovaries than in premenopausal status [44] and was higher in the follicular fluid of fertilized follicles than in the fluid from non-fertilized follicles [22], indicating its importance in gametogenesis and fertilization. The *catalase* protects the genome of mouse oocytes from oxidative damage during meiotic maturation and its activity in ovarian granulosa and theca cells are known to increase during ovarian development and luteinization [22]. In addition, the cooperative effect of *gpx* and catalase has been investigated in other studies [45,46,47]. For instance, the upregulation of *gpx* supported catalase activity in the presence of hydrogen peroxide, thereby inhibiting ROS-induced cell death [46]. Thus, the increase in the levels of *Gpx1* and catalase levels are thought to induce qualitative improvement of in vivo matured oocytes and enhancement of implantation rates by promoting antioxidant capacity in the ovaries of AMA mice.

To optimize the efficacy of treatment, it is necessary to consider dosage, dose frequency, interval, and the site of injection. Injection interval-dependent effects of cell therapy have been scarcely studied; however, there are studies that have reported on the optimal efficacy of stem cell administration during high injection frequencies. For instance, a double injection of human MSCs increased the survival of injected cells and their paracrine effects in a critical limb ischemia mouse model when compared to a single injection containing the same dosage of cells [48]. A study on an acute myocardial infarction rat model also indicated that multiple injections of rat MSCs had greater efficacy than single administration [49]. It is consistent with our results that the expression levels of *gpx1* and catalase in ovaries was significantly enhanced in the group treated with a high frequency of ASC-CM injection regardless of their age. Therefore, the powerful anti-oxidative effect of human ASC-CM had a great impact on the ovaries of 6-month-old mice, especially in the group with high frequency of administration, leading to an enhanced number of implanted fetuses as an indicator of in vivo embryo development.

## 5. Conclusions

In accordance with our results, the antioxidant ability of human ASC-CMs was demonstrated and its anti-oxidative effect on ovarian aging was maximized by higher frequency of administration, thereby improving in vitro and in vivo embryo development in maternally aged mice. Therefore, ASC-CMs could be applied as an antioxidant intervention against age-related female infertility and adjuvant for ART in AMA.

## Figures and Tables

**Table 1:** Primer sequences for real-time polymerase chain reaction.

Genes	GenBank Accession No.	Primer Sequences (5’–3’)	References
*18S rRNA*	NR_003278.3	F: ACCGCGGTTCTATTTTGTTG	[28,29]
		R: CCCTCTTAATCATGGCCTCA	
*Bax*	NM_007527.3	F: ACCAAGAAGCTGAGCGAGTG	[23,24]
		R: TGCAGCTCCATATTGCTGTC	
*Bcl2*	NM_009741.5	F: ATGATAACCGGGAGATCGTG	[24,30]
		R: AGCCCCTCTGTGACAGCTTA	
*Caspase3*	NM_001284409.1	F: TGTCATCTCGCTCTGGTACG	[24,25]
		R: ATTTCAGGCCCATGAATGTC	
*Sod2*	NM_013671.3	F: CTGTCTTCAGCCACACCAGA	[20,21,22]
		R: CTGCTCTTCCAAAGGTCCTG	
*Gpx1*	NM_008160.6	F: CCGACCCCAAGTACATCATT	[20,21,22]
		R: CCCACCAGGAACTTCTCAAA	
Catalase	NM_009804.2	F: TTGACAGAGAGCGGATTCCT	[20,21,22]
		R: TCTGGTGATATCGTGGGTGA	

## Supplementary Materials

The following are available online at https://www.mdpi.com/2076-2615/10/6/978/s1, Table S1: Information pertaining to the mice used in in vitro embryo development evaluation, Table S2: Information pertaining to the mice used in in vivo embryo development evaluation.
